# Shoulder work-related musculoskeletal disorders and related factors of workers in 15 industries of China: a cross-sectional study

**DOI:** 10.1186/s12891-022-05917-2

**Published:** 2022-11-03

**Authors:** Jing Liang, Ning Jia, Feiruo Zhang, HuadongZhang, Ruijie Ling, Yimin Liu, Gang Li, Dongxia Li, Yan Yin, Hua Shao, Hengdong Zhang, Bing Qiu, Xinglin Fang, Dayu Wang, Qiang Zeng, Jianchao Chen, Danying Zhang, Liangying Mei, Yongquan Liu, Jixiang Liu, Chengyun Zhang, Tianlai Li, Yu Li, Huaiying Tao, Huan Luo, Rugang Wang, Zhongxu Wang

**Affiliations:** 1 Institute of Occupational Health, Beijing Center for Diseases Prevention and Control, Beijing, 100020 China; 2 Department of Occupational Protection and Ergonomics, National Institute of Occupational Health and Poison Control, Chinese Center for Disease Control and Prevention, Beijing, 100050 China; 3 Chongqing for Disease Control and Prevention, Chongqing, 400042 China; 4 Hubei Provincial Hospital of Integrated Chinese & Western Medicine, Wuhan, 430015 China; 5 Guangzhou Twelfth People’s Hospital Affiliated to Guangzhou Medical University, Guangzhou, 510400 China; 6 Liaoning Provincial Health Supervision Center, Shenyang, 110005 China; 7 Guizhou Province Occupational Disease Prevention and Control Hospital, uiyang, 550006 China; 8 Shanghai Center for Disease Control and Prevention, Shanghai, 200336 China; 9 Shandong Academy of Occupational Health and Occupational Medicine, Jinan, 250062 China; 10 Jiangsu Provincial Center for Disease Control and Prevention, Nanjing, 210009 China; 11 Civil Aviation Medicine Center, Civil Aviation Administration of China, Beijing, 100025 China; 12 Zhejiang Provincial Center for Disease Control and Prevention, Hangzhou, 310051 China; 13 Tianjin Occupational Diseases Precaution and Therapeutic Hospital (Tianjin Workers’ Hospital), Tianjin, 300011 China; 14 Tianjin Center for Diseases Control and Prevention, Tianjin, 300011 China; 15 Fujian Province Occupational Disease and Chemical Poisoning Prevention and Control Center, Fuzhou, 350025 China; 16 Guangdong Province Hospital for Occupational Disease Prevention and Treatment, Guangzhou, 510300 China; 17 Hubei Provincial Center for Disease Control and Prevention, Wuhan, 430079 China; 18 Institute of Occupational Medicine of Jiangxi, Nanchang, 330006 China; 19 Ningxia Hui Autonomous Region Center for Disease Control and Prevention, Yinchuan, 750004 China; 20 Sichuan Provincial Center for Diseases Control and Prevention, Chengdu, 610041 China; 21 Shanxi Provincial Center for Diseases Control and Prevention, Xian, 710054 China

**Keywords:** Work-related musculoskeletal disorders (WMSD), Shoulder pain, Related factor

## Abstract

**Background:**

Changes in modern industrial production practices can easily lead to shoulder work-related musculoskeletal disorders (WMSD). The current reports on shoulder WMSD are limited to some industries are less well studied, and the sample size is usually small. This study aimed to describe the prevalence and severity of shoulder WMSD in a large sample of Chinese workers from 15 industries, analyze the possible correlations with sociodemographic and work-related variables, and compare the differences between industries.

**Methods:**

A cross-sectional study was conducted among a sample of 55,749 participants from 252 enterprises in 15 industries throughout China. A Chinese version of the musculoskeletal disease questionnaire was used to collect the demographic factors, shoulder symptoms in past 12 months, and work-related factors including posture-related factors, repetition, vibration, work organization, job control, and environmental factors as independent variables. Descriptive statistics were used, and the binary logistic regression analysis was performed to explore the association between shoulder WMSD and potential demographic and work-related factors.

**Results:**

Nearly 35.5% of participants reported shoulder pain and discomfort in the previous 12 months. Biopharmaceutical manufacturing (56.2%), medical services (54.4%), and aviation services (50.1%) were the three industries with the highest prevalence of shoulder WMSD. The pain score of aviation services workers was the highest. The related factors for shoulder WMSD varied among the different industries.

**Conclusion:**

Our study found a relatively high prevalence of shoulder WMSD in China. There were large differences in the prevalence of shoulder WMSD among industries, and the related factors were particular to each industry. Such information is useful to help occupational health practitioners and policymakers conduct preventive programs to reduce shoulder disorders in these working populations.

## Background

Work-related musculoskeletal disorders (WMSD), which the US Department of Labor defines as injuries or disorders of the muscles, nerves, tendons, joints, cartilage, and spinal discs associated with exposure to risk factors in the workplace [1], are the second most common work-related problem worldwide [2].

With the development of industrial science and technology and changes in production practices, forced posture movements of low-load, rapid rhythms and high repetition for long duration have become a primary feature of modern industry. These work practices can easily lead to local muscle fatigue, and in severe cases, local musculoskeletal disease. An epidemiological study found that one of the most common sites of WMSD among workers was the shoulder [3]. Previous research on shoulder WMSD has focused on medical staff [4], such as nurses [5], dentists [6], and surgeons [7]. Although there are some studies on shoulder WMSD in other industries, such as aviation services [8] and vegetable greenhouses [9], more information is needed. In addition, there are no reports of the prevalence of shoulder WMSD in automobile 4S workplaces (4S indicates Sales, Spare parts, Service, and Survey).

According to several previous studies [10–12], demographic factors (e.g., age, gender, body mass index, and education level) are associated with WMSD, and occupational factors such as posture-related factors, work organization, and environmental factors can significantly contribute to pain. Wang et al. [1] reported that years of work and perceived health status increased the prevalence of shoulder symptoms. Seidler et al. [13] identified a dose–response relationship between specific shoulder diseases and hands at/above shoulder level, repetitive movements, forceful work, and hand/arm vibrations.

The current study aimed to determine the prevalence and severity of shoulder WMSD in Chinese workers from various industries and investigated the possible correlations with sociodemographic and work-related variables. These findings could help occupational health practitioners and policymakers conduct specific interventions to reduce shoulder disorders in these working populations.

## Methods

### Survey participants

The survey was carried out in the north, the east, the central, the south, the southwest, the northwest, and the northeast 7 regions of China, and the participants were from 15 industries, including automobile manufacturing, shoemaking, shipbuilding, medical services, construction, electronic equipment manufacturing, animal husbandry, vegetable greenhouses, and automobile 4S workplaces. In each industry, 1 ~ 2 large enterprises, 2 ~ 4 medium-sized enterprises, and 5 ~ 7 small enterprises were selected, according to national classification standard for small and medium-sized enterprises [14]. A total of 252 enterprises were included in the study.

All employees of the selected enterprises participated in the survey. Full-time workers with more than 1 year of employment at the time of investigation were recruited as study participants. Workers who had musculoskeletal disorders caused by non-work-related factors, such as trauma, infectious diseases, rheumatic diseases, malignant tumors, congenital diseases, were excluded.

### Survey methods

To assess the prevalence of shoulder WMSD in China, a Chinese version of the musculoskeletal disease questionnaire was used, which was based on the Nordic Musculoskeletal Questionnaire (NMQ) [15, 16]. The validity and reliability of the Chinese version of NMQ have been previously validated in Chinese occupational groups [17, 18]. An electronic version of the questionnaire was revised by the Institute of Occupational Health and Poisoning, China Center for Disease Control and Prevention.

The survey adopted the 1: N face-to-face filling method, that is, a trained investigator instructed 2 ~ 4 participants to fill in the form themselves using the mobile client. The electronic version of the questionnaire included logic error correction processing. Finally, 55,749 valid questionnaires were received, and the effective recovery rate was 100%.

### Questionnaire

The questionnaire consisted of three sections: demographic factors, musculoskeletal symptoms, and work-related factors.

The first section gathered information on gender, date of birth, years of work, marital status, highest education level, physical activity, monthly income, and perceived health status.

The second section of the questionnaire captured information on musculoskeletal symptoms. The participants were asked whether, in the past 12 months, they had experienced discomfort, numbness, pain, or limited activity in the shoulder lasting more than 24 hours or that did not resolve after their workday [19]. If they had any positive symptoms, they were asked to answer supplementary questions about their shoulder-related pain frequency (more than a week in every month/less than a week in every month/more than a week but not in every month/less than a week but not in every month), accumulated duration in the past year (1 ~ 7 days/8 ~ 30 days/more than 30 days but not every day/almost every day), and self-feeling pain level (score 1 ~ 10, with score 1 for mild and score 10 for unbearable).

The third section of the questionnaire focused on work-related factors. There were 21 items and all the detailed descriptions of independent variables in Table 1.

**Table 1:** Checklist of work-related factors

Standing for a long time	Seldom or never/sometimes/ often/frequent
Sitting for a long time	Seldom or never/sometimes/ often/frequent
Squatting for a long time	Seldom or never/sometimes/ often/frequent
Uncomfortable posture	Seldom or never/sometimes/ often/frequent
Heavy physical labor with the upper limbs or hands	Seldom or never/sometimes/ often/frequent
Use vibration tools when working	Seldom or never/sometimes/ often/frequent
Lifting objects weighing more than 5 kg	Seldom or never/sometimes/ often/frequent
Driving cars	Seldom or never/sometimes/ often/frequent
Hand position during operation	Shoulder or below shoulder level/ above shoulder level
Do the same work almost every day	Yes/no
Repeated operation numerous times per minute	Yes/no
Take turns with coworkers	Yes/no
Outdoors	Yes/no
Deal with customers, patients and the public	Yes/no
Feeling cold, wind or temperature change at work	Yes/no
Often work overtime	Yes/no
Enough rest time	Yes/no
Self-decision when to start and finish work	Yes/no
Self-decision when to take a break	Yes/no
Shortage of staff in the work department	Yes/no
Substitute for colleagues	Yes/no

### Data analysis

The data obtained from the questionnaire were analyzed using SPSS version 21.0 (IBM Corp., Armonk, NY, USA). The Kolmogorov-Smirnov test was used to determine the normality of the data. Continuous data accorded with normal distribution were expressed as the mean ± standard deviation; otherwise, they were presented as median (Q10, Q90). Categorical variables are presented as numbers and percentages. Chi-square tests were used to determine differences among multiple groups. Binary logistic regression models were then developed to estimate associations between variables and shoulder WMSD. The variables considered in the analysis included 8 demographic factors and 21 work-related factors. Independent models were constructed for each industry. For those industries with small sample size, such as animal husbandry, automobile 4S workplaces, biopharmaceutical manufacturing, petrochemical industry, vegetable greenhouses and toy manufacturing, chi-squared tests were conducted first to select variables (*P* < 0.1). The fit of the models was assessed by the Hosmer–Lemeshow test. The odds ratios (ORs) and respective 95% confidence intervals (95% CI) were calculated as measures of association between the dependent variable and independent variables. The significance level was set as 0.05.

## Results

### Demographics characteristics of the participants

In total, 36,155 (64.9%) participants were male. The mean age of the participants was 32 (23, 47) years, and a majority were 20 ~ 40 years old (*n* = 42,293, 75.9%). The average length of employment was 6 (2, 11) years, and a majority had been employed for 1 ~ 15 years (*n* = 45,881, 82.3%).

### Prevalence of shoulder WMSD

A total of 19,779 participants reported work-related musculoskeletal pain or discomfort in the shoulder in the previous 12 months; the prevalence of shoulder WMSD was 35.5%. Biopharmaceutical manufacturing, medical services, and aviation services had the highest prevalence of shoulder WMSD, all of which were higher than 50%. By contrast, the prevalence of shoulder WMSD in the petrochemical industry and construction industry was less than 20%.

The number and proportion of participants in each industry were presented in Table 2. The Chi-square test showed significant differences in the prevalence of shoulder WMSD among the industries (*p* < 0.05).

**Table 2:** Prevalence rate and pain level of shoulder WMSDs of participants in 15 industries

Industry	N.	Proportion(%)	The shoulder musculoskeletal symptoms in the past 12 months	
			No. of cases	Prevalence, %
**Animal husbandry**	247	0.44	55	22.3
**Shipbuilding**	3353	6.01	1137	33.9
**Electronic equipment manufacturing**	7780	13.96	2680	34.4
**Furniture manufacturing**	4409	7.91	1138	25.8
**Construction**	1357	2.43	248	18.3
**Coal mining and washing industry**	1458	2.62	509	34.9
**Aviation services**	1314	2.36	658	50.1
**Automobile 4S workplaces**	604	1.08	229	37.9
**Automobile manufacturing**	20,806	37.32	6757	32.5
**Biopharmaceutical manufacturing**	242	0.43	136	56.2
**Petrochemical industry**	148	0.27	17	11.5
**Vegetable greenhouses**	243	0.44	64	26.3
**Toy manufacturing**	314	0.56	146	46.5
**Medical services**	6564	11.77	3571	54.4
**Shoemaking**	6910	12.39	2434	35.2
**Total**	55,749	100	19,779	35.5

### Severity of shoulder WMSD

Shoulder WMSD was graded from mild (score of 1) to unbearable (score of 10). The pain score of flight attendants was the highest. Detailed data on the frequency of occurrence and cumulative duration of shoulder symptoms were presented in Table 3.

**Table 3:** Severity of shoulder WMSDs among participants in 15 industries

Industry	Pain level, M(Q10, Q90)	Frequency of occurrence, N (%)				Cumulative duration of symptoms, N (%)			
		More than a week in every month	Less than a week in every month	More than a week but not in every month	Less than a week but not in every month	1 ~ 7 days	8 ~ 30 days	> 30 days, but not everyday	Almost every day
**Animal husbandry**	4 (2, 6)	6 (10.9)	10 (18.2)	24 (43.6)	15 (27.3)	14 (25.5)	24 (43.6)	14 (25.5)	3 (5.5)
**Shipbuilding**	4 (2, 7)	271 (23.8)	315 (27.7)	129 (11.3)	422 (37.1)	453 (39.8)	334 (29.4)	279 (24.5)	71 (6.2)
**Electronic equipment manufacturing**	5 (3, 7)	661 (24.7)	878 (32.8)	249 (9.3)	892 (33.3)	899 (33.5)	704 (26.3)	823 (30.7)	254 (9.5)
**Furniture manufacturing**	5 (2, 7)	275 (24.2)	285 (25.0)	134 (11.8)	444 (39.0)	562 (49.4)	233 (20.5)	249 (21.9)	94 (8.3)
**Construction**	5 (3, 7)	36 (14.5)	82 (33.1)	33 (13.3)	97 (39.1)	77 (31.0)	80 (32.3)	82 (33.1)	9 (3.6)
**Coal mining and washing industry**	5 (3, 8)	180 (35.4)	155 (30.5)	66 (13.0)	108 (21.2)	170 (33.4)	125 (24.6)	160 (31.4)	54 (10.6)
**Aviation services**	6 (4, 8)	120 (18.2)	251 (38.1)	62 (9.4)	225 (34.2)	200 (30.4)	199 (30.2)	219 (33.3)	40 (6.1)
**Automobile 4S workplaces**	5 (2, 7)	57 (24.9)	60 (26.2)	15 (6.6)	97 (42.4)	75 (32.8)	57 (24.9)	84 (36.7)	13 (5.7)
**Automobile manufacturing**	5 (3, 7)	1738 (25.7)	2050 (30.3)	652 (9.6)	2317 (34.3)	2461 (36.4)	1649 (24.4)	1895 (28.0)	752 (1.1)
**Biopharmaceutical manufacturing**	5 (3, 7)	27 (19.9)	53 (39.0)	13 (9.6)	43 (31.6)	26 (19.1)	34 (25.0)	67 (49.3)	9 (6.6)
**Petrochemical industry**	4 (1, 6)	4 (23.5)	2 (11.8)	1 (5.9)	10 (58.8)	8 (47.1)	5 (29.4)	3 (17.6)	1 (5.9)
**Vegetable greenhouses**	4 (3, 7)	11 (17.2)	20 (31.3)	15 (23.4)	18 (28.1)	7 (10.9)	22 (34.4)	30 (46.9)	5 (7.8)
**Toy manufacturing**	4 (4, 7)	50 (34.2)	45 (30.8)	11 (7.5)	40 (27.4)	52 (35.6)	34 (23.3)	39 (26.7)	21 (14.4)
**Medical services**	5 (3, 7)	951 (26.6)	1157 (32.4)	371 (10.4)	1092 (30.6)	1081 (30.3)	974 (27.3)	1161 (32.5)	355 (9.9)
**Shoemaking**	4 (2, 7)	598 (24.6)	671 (27.6)	269 (11.1)	896 (36.8)	873 (35.9)	623 (25.6)	671 (27.6)	267 (11.0)
**Total**	5 (3, 7)	4985 (25.2)	6034 (30.5)	2044 (10.3)	6716 (34.0)	6958 (35.2)	5097 (25.8)	5776 (29.2)	1948 (9.8)

#### The gender and age characteristics of shoulder WMSD

Generally speaking, the prevalence of shoulder WMSD in women was higher than that in men according to Chi-square test results. There were significant differences in gender in most industries except animal husbandry, coal mining and washing industry, biopharmaceutical manufacturing, and toy manufacturing. A significant difference in the prevalence of WMSDs was observed between different age groups. Detailed data were presented in Table 4.

**Table 4:** Gender and age characteristics of shoulder WMSD among participants in 15 industries

Industry	Gender, N. of cases/N. (%)			Age (years), N. of cases/N. (%)					
	Male	Female	***p***	< 25	25-	35-	45-	55-	***p***
**Animal husbandry**	30/157 (19.1)	25/90 (27.8)	0.079	2/6 (33.3)	34/148 (23.0)	10/60 (16.7)	9/26 (34.6)	0/7 (0)	0.211
**Shipbuilding**	899/2758 (32.6)	238/596 (40.0)	< 0.05	28/444 (6.3)	387/1545 (25.0)	367/831 (44.2)	300/428 (70.1)	55/105 (52.4)	< 0.05
**Electronic equipment manufacturing**	875/3787 (23.1)	1805/3993 (45.2)	< 0.05	102/1329 (7.7)	895/3727 (24.0)	923/1684 (54.8)	628/806 (77.9)	132/234 (56.4)	< 0.05
**Furniture manufacturing**	772/3126 (24.7)	366/1283 (28.5)	< 0.05	156/658 (23.7)	734/2352 (31.2)	209/989 (21.1)	32/310 (10.3)	7/100 (7.0)	< 0.05
**Construction**	213/1238 (17.2)	35/119 (29.4)	< 0.05	14/240 (5.8)	141/748 (18.9)	70/286 (24.5)	19/66 (28.8)	4/17 (23.5)	< 0.05
**Coal mining and washing industry**	484/1399 (34.6)	25/59 (42.2)	0.264	47/187 (25.1)	237/646 (46.6)	147/439 (33.5)	73/155 (47.1)	5/31 (16.1)	< 0.05
**Aviation services**	66/293 (22.5)	592/1021 (58.0)	< 0.05	79/165 (47.9)	315/660 (47.7)	204/373 (54.7)	52/100 (52)	8/16 (50)	0.277
**Automobile 4S workplaces**	173/545 (31.7)	56/59 (94.9)	< 0.05	29/95 (30.5)	116/350 (33.1)	74/134 (55.2)	9/21 (42.9)	1/4 (25.0)	< 0.05
**Automobile manufacturing**	6023/19306 (31.2)	734/1500 (48.9)	< 0.05	1050/4359 (24.1)	3227/10012 (32.2)	1963/4445 (44.2)	440/1446 (30.4)	77/544 (14.2)	< 0.05
**Biopharmaceutical manufacturing**	55/109 (50.5)	81/133 (60.9)	0.119	26/31 (83.9)	70/120 (58.3)	31/70 (44.3)	7/15 (46.7)	2/6 (33.3)	< 0.05
**Petrochemical industry**	13/142 (9.2)	4/6 (66.7)	< 0.05	0/7 (0)	9/84 (10.7)	3/38 (7.9)	4/15 (26.7)	1/4 (25.0)	0.235
**Vegetable greenhouses**	33/154 (21.4)	31/89 (34.8)	< 0.05	7/14 (50.0)	36/133 (27.1)	15/68 (22.1)	6/24 (25.0)	0/4 (0)	0.187
**Toy manufacturing**	43/109 (39.4)	103/205 (50.2)	0.075	8/8 (100)	62/127 (48.8)	60/127 (47.2)	15/42 (35.7)	1/10 (10.0)	< 0.05
**Medical services**	398/826 (48.2)	3173/5738 (55.3)	< 0.05	221/428 (51.6)	1440/2275 (63.3)	1090/2038 (53.5)	642/1527 (42.0)	178/296 (60.1)	< 0.05
**Shoemaking**	580/2206 (26.3)	1854/4704 (39.4)	< 0.05	69/342 (20.2)	1345/2526 (53.2)	587/2233 (26.3)	375/1655 (22.7)	58/154 (37.7)	< 0.05
**Total**	10,657 (29.5)	9122 (46.6)	< 0.05	1838 (22.1)	29,048 (35.5)	5753 (41.6)	2611 (39.3)	529 (34.5)	< 0.05

### Related factors associated with shoulder WMSD

The related factors with statistical significances in binary logistic regression models were presented in Table 5.

**Table 5:** The OR (95% CI) of related factors for shoulder WMSD among participants in 15 industries

Industry	Factors	Categories	ORs	95% CI	
				Lower	Upper
**Animal husbandry**	Repeated operation numerous times per minute	No	1.00	–	
		Yes	2.01	1.03	3.91
	Feeling cold, wind or temperature change at work	No	1.00	–	
		Yes	2.74	1.18	6.39
**Shipbuilding**	Gender	Male	1.00	–	
		Female	1.79	1.36	2.35
	Age		1.08	1.05	1.12
	Physical activity	No	1.00	–	
		occasional	1.71	1.10	2.66
		2-3 times/month	1.81	1.18	2.77
		1-2 times/week	2.22	1.22	4.02
		> 2 times/week	1.95	1.15	3.30
	Perceived health status	perfect	1.00	–	
		fine	1.91	1.59	2.31
		poor	3.30	2.23	4.90
		very poor	2.02	0.76	5.37
	Heavy physical labor with the upper limbs or hands	seldom/never	1.00	–	
		sometimes	1.19	0.84	1.69
		often	1.41	1.00	1.99
		frequent	2.24	1.55	3.24
	Uncomfortable posture	seldom/never	1.00	–	
		sometimes	1.24	0.98	1.57
		often	1.87	1.39	2.51
		frequent	2.37	1.60	3.51
	Do the same work almost every day	No	1.00	–	
		Yes	1.55	1.15	2.08
	Feeling cold, wind or temperature change at work	No	1.00	–	
		Yes	1.33	1.07	1.65
	Enough rest time	No	1.00	–	
		Yes	0.72	0.59	0.87
	Self-decision when to start and finish work	No	1.00	–	
		Yes	0.78	0.61	0.99
**Electronic equipment manufacturing**	Gender	Male	1.00	–	
		Female	2.22	1.91	2.58
	Age		1.11	1.09	1.14
	Length of employment (years)	< 2	1.00	–	
		2 ~ 6	1.13	0.94	1.36
		7 ~ 11	1.56	1.16	2.10
		≥12	1.76	1.09	2.85
	Perceived health status	perfect	1.00	–	
		fine	1.92	1.68	2.20
		poor	2.74	2.11	3.57
		very poor	3.82	1.98	7.36
	Sitting for a long time	seldom/never	1.00	–	
		sometimes	1.43	1.18	1.73
		often	2.80	2.28	3.44
		frequent	3.73	2.91	4.77
	Lifting object weighing more than 5 kg	seldom/never	1.00	–	
		sometimes	1.18	1.01	1.38
		often	1.70	1.37	2.11
		frequent	1.34	0.97	1.84
	Heavy physical labor with the upper limbs or hands	seldom/never	1.00	–	
		sometimes	1.19	1.00	1.43
		often	1.35	1.12	1.63
		frequent	1.82	1.46	2.28
	Uncomfortable posture	seldom/never	1.00	–	
		sometimes	1.47	1.27	1.70
		often	2.26	1.75	2.93
		frequent	2.65	1.78	3.94
	Repeated operation numerous times per minute	No	1.00	–	
		Yes	1.70	1.48	1.94
	Take turns with coworkers	No	1.00	–	
		Yes	0.80	0.70	0.90
	Enough rest time	No	1.00	–	
		Yes	0.63	0.56	0.72
**Furniture manufacturing**	Gender	Male	1.00	–	
		Female	1.32	1.09	1.59
	Age		0.97	0.94	0.99
	Education	junior high school	1.00	–	
		senior high school	1.23	1.04	1.47
		Junior College	1.62	1.21	2.18
		bachelor degree or above	1.84	1.15	2.93
	Perceived health status	perfect	1.00	–	
		fine	2.02	1.73	2.37
		poor	3.01	2.15	4.22
		very poor	1.44	0.70	2.94
	Heavy physical labor with the upper limbs or hands	seldom/never	1.00	–	
		sometimes	1.10	0.78	1.56
		often	1.17	0.83	1.63
		frequent	1.57	1.09	2.25
	Uncomfortable posture	seldom/never	1.00	–	
		sometimes	1.55	1.30	1.85
		often	2.60	1.98	3.43
		frequent	2.14	1.43	3.19
	Repeated operation numerous times per minute	No	1.00	–	
		Yes	1.34	1.12	1.60
	Do the same work almost every day	No	1.00	–	
		Yes	1.87	1.35	2.59
	Outdoors	No	1.00	–	
		Yes	0.58	0.37	0.92
	Deal with customers, patients and the public	No	1.00	–	
		Yes	1.48	1.06	2.06
	Feeling cold, wind or temperature change at work	No	1.00	–	
		Yes	1.21	1.00	1.45
	Enough rest time	No	1.00	–	
		Yes	0.69	0.58	0.81
	Shortage of staff in the work department	No	1.00	–	
		Yes	1.25	1.05	1.48
**Construction**	Gender	Male	1.00	–	
		Female	2.43	1.34	4.40
	Age		1.09	1.02	1.16
	Lifting object weighing more than 5 kg	seldom/never	1.00	–	
		sometimes	1.24	0.66	2.32
		often	4.05	2.03	8.08
		frequent	3.50	1.52	8.06
	Heavy physical labor with the upper limbs or hands	seldom/never	1.00	–	
		sometimes	2.41	1.07	5.43
		often	2.92	1.27	6.73
		frequent	2.96	1.17	7.47
	Uncomfortable posture	seldom/never	1.00	–	
		sometimes	2.47	1.53	4.00
		often	2.66	1.39	5.09
		frequent	3.67	0.99	13.56
	Enough rest time	No	1.00	–	
		Yes	0.66	0.44	0.97
	Substitute for colleagues	No	1.00	–	
		Yes	2.03	1.27	3.26
**Coal mining and washing industry**	Gender	Male	1.00	–	
		Female	2.37	1.25	4.51
	Perceived health status	perfect	1.00	–	
		fine	1.84	1.38	2.45
		poor	2.90	1.87	4.50
		very poor	4.71	1.94	11.40
	Feeling cold, wind or temperature change at work	No	1.00	–	
		Yes	1.76	1.29	2.40
	Often work overtime	No	1.00	–	
		Yes	1.38	1.03	1.85
	Shortage of staff in the work department	No	1.00	–	
		Yes	1.42	1.07	1.87
**Aviation services**	Gender	Male	1.00	–	
		Female	3.10	1.97	4.88
	Perceived health status	perfect	1.00	–	
		fine	2.44	1.59	3.74
		poor	3.25	1.93	5.49
		very poor	3.55	1.41	8.95
	Uncomfortable posture	seldom/never	1.00	–	
		sometimes	2.11	1.43	3.11
		often	2.16	1.36	3.43
		frequent	4.55	2.50	8.26
**Automobile 4S workplaces**	Age		1.04	1.01	1.08
	Uncomfortable posture	seldom/never	1.00	–	
		sometimes	1.94	1.00	3.76
		often	3.27	1.46	7.33
		frequent	3.69	1.27	10.69
	Enough rest time	No	1.00	–	
		Yes	0.44	0.26	0.75
	Shortage of staff in the work department	No	1.00	–	
		Yes	1.87	1.13	3.08
**Automobile manufacturing**	Gender	Male	1.00	–	
		Female	2.11	1.86	2.41
	Age		1.03	1.02	1.05
	Length of employment (years)	< 2	1.00	–	
		2 ~ 6	1.39	1.26	1.54
		7 ~ 11	1.54	1.33	1.78
		≥12	1.61	1.30	1.98
	Education	junior high school	1.00	–	
		senior high school	0.98	0.85	1.12
		Junior College	1.06	0.92	1.23
		bachelor degree	1.38	1.13	1.67
		master degree or above	1.67	1.10	2.53
	Perceived health status	perfect	1.00	–	
		fine	1.67	1.56	1.80
		poor	2.33	2.06	2.64
		very poor	2.35	1.74	3.16
	Lifting object weighing more than 5 kg	seldom/never	1.00	–	
		sometimes	1.05	0.97	1.14
		often	1.25	1.12	1.39
		frequent	1.19	1.05	1.34
	Sitting for a long time	seldom/never	1.00	–	
		sometimes	1.02	0.93	1.12
		often	1.59	1.40	1.80
		frequent	1.50	1.30	1.74
	Heavy physical labor with the upper limbs or hands	seldom/never	1.00	–	
		sometimes	1.03	0.90	1.16
		often	1.23	1.08	1.40
		frequent	1.46	1.28	1.66
	Uncomfortable posture	seldom/never	1.00	–	
		sometimes	1.51	1.40	1.64
		often	2.04	1.82	2.29
		frequent	2.62	2.28	3.01
	Repeated operation numerous times per minute	No	1.00	–	
		Yes	1.34	1.25	1.43
	Do the same work almost every day	No	1.00	–	
		Yes	1.13	1.02	1.26
	Take turns with coworkers	No	1.00	–	
		Yes	0.91	0.85	0.98
	Outdoors	No	1.00	–	
		Yes	0.80	0.69	0.92
	Deal with customers, patients and the public	No	1.00	–	
		Yes	1.19	1.04	1.37
	Feeling cold, wind or temperature change at work	No	1.00	–	
		Yes	1.16	1.08	1.25
	Often work overtime	No	1.00	–	
		Yes	1.29	1.20	1.40
	Enough rest time	No	1.00	–	
		Yes	0.67	0.62	0.72
	Shortage of staff in the work department	No	1.00	–	
		Yes	1.13	1.05	1.21
**Biopharmaceutical manufacturing**	Age		0.93	0.89	0.97
**Petrochemical industry**	Monthly income (RMB)	≤1000	1.00	–	
		1001 ~ 3000	0.02	0.00	0.29
		3001 ~ 5000	0.04	0.00	0.72
**Vegetable greenhouses**	Gender	Male	1.00	–	
		Female	2.10	1.13	3.91
**Toy manufacturing**	Gender	Male	1.00	–	
		Female	1.98	1.02	3.83
	Age		0.95	0.92	0.98
	Take turns with coworkers	No	1.00	–	
		Yes	0.58	0.34	0.99
	Perceived health status	perfect	1.00	–	
		fine	2.86	1.67	4.89
		poor	2.61	0.59	11.60
	Shortage of staff in the work department	No	1.00	–	
		Yes	2.34	1.26	4.37
**Medical services**	Gender	Male	1.00	–	
		Female	1.66	1.38	1.99
	Age		0.98	0.96	1.00
	Length of employment (years)	< 2	1.00	–	
		2 ~ 6	1.25	1.01	1.56
		7 ~ 11	1.50	1.15	1.97
		≥12	1.54	1.07	2.21
	Perceived health status	perfect	1.00	–	
		fine	1.65	1.44	1.90
		poor	2.50	2.04	3.06
		very poor	2.07	1.32	3.24
	Sitting for a long time	seldom/never	1.00	–	
		sometimes	1.04	0.89	1.20
		often	1.57	1.31	1.89
		frequent	2.18	1.75	2.72
	Heavy physical labor with the upper limbs or hands	seldom/never	1.00	–	
		sometimes	1.11	0.92	1.34
		often	1.29	1.07	1.56
		frequent	1.36	1.11	1.66
	Uncomfortable posture	seldom/never	1.00	–	
		sometimes	1.75	1.52	2.02
		often	2.76	2.30	3.32
		frequent	3.01	2.33	3.90
	Do the same work almost every day	No	1.00	–	
		Yes	1.33	1.07	1.66
	Take turns with coworkers	No	1.00	–	
		Yes	0.83	0.73	0.94
	Feeling cold, wind or temperature change at work	No	1.00	–	
		Yes	1.17	1.02	1.34
	Often work overtime	No	1.00	–	
		Yes	1.22	1.08	1.37
	Enough rest time	No	1.00	–	
		Yes	0.77	0.68	0.87
**Shoemaking**	Gender	Male	1.00	–	
		Female	1.73	1.48	2.03
	Length of employment (years)	< 2	1.00	–	
		2 ~ 6	1.24	1.02	1.53
		7 ~ 11	1.51	1.14	2.00
		≥12	1.15	0.75	1.76
	Education	junior high school	1.00	–	
		senior high school	1.22	1.05	1.41
		Junior College	2.80	2.01	3.91
		bachelor degree	3.34	1.77	6.32
	Physical activity	seldom/never	1.00	–	
		sometimes	1.18	1.04	1.34
		often	1.25	0.90	1.73
		frequent	1.38	1.07	1.77
	Perceived health status	perfect	1.00	–	
		fine	1.88	1.67	2.12
		poor	3.52	2.56	4.85
		very poor	2.24	1.20	4.19
	Standing for a long time	seldom/never	1.00	–	
		sometimes	1.26	1.08	1.46
		often	1.52	1.25	1.84
		frequent	1.31	1.03	1.66
	Sitting for a long time	seldom/never	1.00	–	
		sometimes	0.88	0.74	1.05
		often	1.34	1.11	1.62
		frequent	1.63	1.31	2.04
	Heavy physical labor with the upper limbs or hands	seldom/never	1.00	–	
		sometimes	1.20	1.01	1.42
		often	1.17	0.99	1.38
		frequent	1.53	1.26	1.88
	Uncomfortable posture	seldom/never	1.00	–	
		sometimes	1.43	1.23	1.67
		often	1.93	1.47	2.54
		frequent	2.21	1.49	3.28
	Repeated operation numerous times per minute	No	1.00	–	
		Yes	1.26	1.10	1.44
	Feeling cold, wind or temperature change at work	No	1.00	–	
		Yes	1.58	1.36	1.83
	Enough rest time	No	1.00	–	
		Yes	0.67	0.59	0.76
	Self-decision when to take a break	No	1.00	–	
		Yes	0.73	0.60	0.89

Several postural factors were found to be associated with musculoskeletal symptoms in the shoulder. ‘Standing for a long time” and/or ‘sitting for a long time” were associated with an increased prevalence of shoulder symptoms in the electronic equipment manufacturing workers, automobile manufacturers, shoemakers, and medical staff. ‘Uncomfortable posture’ and ‘heavy physical labor with the upper limbs or hands’ were associated with an increased prevalence of shoulder symptoms in shipbuilding, electronic equipment manufacturing, furniture manufacturing, construction, automobile manufacturing, shoemaking, and medical services workers.

Repeated work as indicated by ‘do the same work almost every day’ and/or ‘repeated operation numerous times per minute’ was associated with the occurrence of shoulder symptoms in the animal husbandry, shipbuilding, electronic equipment manufacturing, furniture manufacturing, automobile manufacturing, shoemaking, and medical services workers. In electronic equipment workers, construction workers, and automobile manufacturers, “lifting objects weighing more than 5 kg” was a related factor of shoulder WMSD.

Regarding work organization, ‘overtime work’ in medical services workers and ‘staff shortages’ in furniture manufacturing, coal mining and washing, automobile 4S workplaces, automobile manufacturing, and toy manufacturing workers significantly increased the participants’ musculoskeletal symptoms in the shoulder. The employees who were able to have ‘enough rest time’ reported fewer shoulder symptoms in most industries. ‘Self-decision when to start and finish work’ for shipbuilders and ‘self-decision when to take a break’ for shoemakers decreased shoulder symptoms.

Two environmental factors were found to be associated with shoulder WMSD, ‘outdoors’ in furniture manufacturing and automobile manufacturing workers, and ‘feeling cold, wind or temperature changes at work’ in animal husbandry, shipbuilding, furniture manufacturing, coal mining and washing, automobile manufacturing, shoemaking, and medical services workers.

In addition to work-related factors, some personal factors were found to have statistical significance. Beside gender and age, poor perceived health status, high educational level, and the length of employment were associated with reported shoulder WMSD in some industries.

## Discussion

According to our study, the prevalence of shoulder WMSD among participants in the key industries was 35.5%. This prevalence was lower than that found by Renée Govaerts et al. in a meta-analysis covering 7 secondary industries in 13 European countries in which the mean 12-month prevalence of shoulder WMSD was 50% [20]. However, our result was far higher than that reported by the US Bureau of Labor Statistics, which found that the incidence of shoulder WMSD was 11.8% in 201 6[21].

In different industries, the prevalence of shoulder WMSD ranged from11.5 to 56.2%. The three industries with the highest prevalence of shoulder WMSD were biopharmaceutical manufacturing (56.2%), medical services (54.4%), and aviation services (50.1%). The prevalence and the related factors of shoulder WMSD were reported for the first time in shipbuilding, automobile 4S workplaces, and some other industries.

Medical personnel have been found to be vulnerable to WMSD in previous surveys. In China, the prevalence of shoulder WMSD has been reported for different types of medical staff, including dentists (92.2%) [22], ultrasound department staff (87%) [23], obstetrics and gynecology staff (62%) [1], nurses (54.6%) [24], and midwives (42.7%) [25]. In our study, 54.4% of medical staff suffered from shoulder pain in the past 12 months. The following factors were found to be associated with shoulder musculoskeletal symptoms: female gender, years of work, perceived health status, sitting for a long time, heavy physical labor with the upper limbs or hands, uncomfortable posture, doing the same work almost every day, feeling cold, wind, or temperature changes at work, and often working overtime.

The previous research in aviation services was not sufficient. Only one study is available, by Rau PP et al., and included a small sample size of Chinese female flight attendants. The study conducted an online questionnaire (46 flight attendants) and semi-structured interviews (16 flight attendants) and identified the following risk factors: working with improperly designed overhead bins and providing food and drink service in an awkward posture [8]. According to our study, female gender, worse perceived health status, and uncomfortable postures were found to be associated with shoulder musculoskeletal problems among the aviation services participants.

The manufacturing industry includes electronic equipment manufacturing, furniture manufacturing, automobile manufacturing, and shoemaking, which are characterized by assembly line operations. The prevalence of shoulder WMSD ranged from 25.8 to 46.5% in these industries, with sitting/standing for a long time, repeated operations, and high hand forces being recognized as important contributing factors. Heavy lifting was a related factor only in electronics manufacturing and automobile manufacturing. Similar prevalence was found in a case-control study conducted in the Spanish automotive manufacturing sector [26], in a cross-sectional study among Iranian hand-woven shoe workers [27], in a cross-sectional study among 931 workers from an electronics factory in Taiwan, China [28], and from self-reported data of shoulder WMSD-injured manufacturing workers in Washington state, USA [29]. Overtime work and staff shortages in the work department significantly increased the participants’ musculoskeletal symptoms in the shoulder. Participants were more likely to be protected from shoulder WMSD if they had sufficient rest and autonomy over their rest.

The prevalence of shoulder WMSD in animal husbandry and vegetable greenhouse workers was 22.3 and 26.3%, respectively. Our results were much lower than that found in 249 vegetable greenhouse farmers in a rural area of Shandong, China (31.7%) [9] and lower than that of the 2-week prevalence of neck/shoulder pain in 518 farmers in the US Midwest (30.8%) [30]. Fethke et al. observed a significant association between milking animals and neck/shoulder pain [30]. Caieiro et al. reported that egg classification, candling, and in vivo vaccination required repetitive shoulder abduction and elbow flexion [31]. Kang et al. found that the main activities in growing greenhouse vegetables (planting seeding, pruning stems and leaves, picking, carrying, shoveling, spraying pesticides, weeding, and fertilizing) resulted in different levels of disorder in the farmers’ musculoskeletal structure [9]. We found that ‘feeling cold, wind or temperature changes at work” was also a related factor for shoulder WMSD in the animal husbandry participants.

The prevalence of shoulder WMSD in construction and petrochemical industry workers was 18.3 and 11.5%, respectively. These data were consistent with studies of construction workers in Hong Kong (19.8%) [32] and India (11.7%) [33]. In our study, ‘female gender’, ‘lifting object weighing more than 5 kg’, ‘heavy physical labor with the upper limbs or hands’, ‘uncomfortable posture’, ‘not enough rest time’, and ‘filling in for colleagues’ were independent related factors for shoulder WMSD in the construction industry workers.

In addition to work-related factors, some demographic and socioeconomic factors, including female gender and age, were significantly associated with the prevalence of shoulder WMSD in most industries. Ning Jia et al. found that female workers had a 1.5 times increased risk of WMSD compared with males, and the highest prevalence was among those aged 35 ~ 45 years [3]. Similarly, the perceived health status was found to be a related factor for shoulder WMSD. In some industries, monthly income (e.g., petrochemical industry), length of employment (e.g., medical services), the highest education level (e.g., automobile manufacturing, furniture manufacturing, and shoemaking), and physical activity (e.g., shoemaking) were also factors that influenced shoulder WMSD. Notably, the demographic characteristics of people differed by position (e.g., age, gender, and education level in frontline workers and administrative staff). It would be necessary to conduct a stratified analysis by gender and age to clarify the risk factors for shoulder WMSD in certain industries.

In addition, the composition of different types of work in various industries may also affect the prevalence of shoulder WMSD in this industry. In the NMQ survey of the 154 production personnel and 24 management personnel of a poultry breeding company, the prevalence of pain in the shoulder was higher among the production employees (47%) compared with administrative employees (38% )[31]. However, in a cross-sectional study using NMQ of 1415 employees (830 frontline workers and 585 other employees) in six industrial companies in Beijing, China, it was found that the prevalence of shoulder pain among frontline employees (15.42%) was significantly lower than that of other employees (19.66%), including office clerks, support staff and technicians [34].

This study has some limitations that should be considered when interpreting the results. First, there could be selection bias in the cross-sectional study because older and less healthy individuals may have left the workforce; reporting bias could also have influenced the results. Second, some participants may have suffered from shoulder musculoskeletal diseases caused by non-work-related factors such as rheumatism and tumors that had not yet been diagnosed, which could have altered the validity of the results. Additionally, some industries related to shoulder WMSD but that are characterized by individual workers rather than organizations (such as hairdressers [35]) were not investigated. Work stress and occupational psychological factors, as well as their interconnections, were also not considered in our study, although earlier studies have linked them to the development of WMSD [25, 36].

## Conclusions

In summary, the prevalence of shoulder WMSD was relatively high in China, with large differences between industries. The three industries with the highest prevalence of shoulder WMSD were biopharmaceutical manufacturing, medical services, and aviation services. This was the first report of such data in some industries. We found that the related factors for shoulder WMSD were different in each industry. Based on the data of this study, further analysis according to different posts (different operations) can give a better explanation to the risk of shoulder WMSD in a certain industry. As a result, it is necessary to take interventional actions based on the specific factors in each industry to reduce the impact of shoulder WMSD. These actions could include improving the work environment, optimizing work organization, and strengthening ergonomics awareness.

## Data Availability

The datasets generated and/or analyzed during the current study are not publicly available due data sharing was not included in the informed consent process, but are available from the corresponding author on reasonable request.

## Abbreviations

WMSD: Work-related musculoskeletal disorders
4S: Sales, Spare parts, Service, and Survey
NMQ: Nordic Musculoskeletal Questionnaire
OR: Odds ratios
CI: confidence intervals
